# Integration of Vehicle–Terrain Interaction and Fuzzy Cost Adaptation for Robust Path Planning

**DOI:** 10.3390/s25175454

**Published:** 2025-09-03

**Authors:** Hongchao Zhang, Qiancheng Zhao, Yinghao Wu, Da Jiang, Xiaole Chen, Xiaoming Liang, Yunlong Sun

**Affiliations:** 1 School of Mechanical Science and Engineering, Huazhong University of Science and Technology, Wuhan 430074, China; d202187005@hust.edu.cn; 2 Northern Vehicle Research Institute, Beijing 100072, China; wyh8896969@126.com (Y.W.); ziangdar@sina.com (D.J.); chenxl4227@163.com (X.C.); a18511981955@gmail.com (X.L.); sunyunlong0124@sina.cn (Y.S.)

**Keywords:** learning-based planning, 3D terrain path planning, task-aware cost adjustment, adaptive planning algorithm

## Abstract

This paper proposes an adaptive path-planning algorithm for unmanned ground vehicles (UGVs) in three-dimensional terrain environments. The algorithm first constructs an interference model between the UGV chassis and the three-dimensional terrain, taking into account the impact of terrain undulations on vehicle driving stability. A dynamic cost-adjustment mechanism for multi-task modes was designed, which introduces a learning model to automatically identify task types and dynamically adjust the weights of various cost factors in path planning accordingly. This paper constructs simulation environments for sparse obstacle scenes and high-density obstacle scenes, respectively, to verify the effectiveness of the path-planning results of the algorithm in different task modes. The experimental results show that the proposed method can generate smoother, safer, and more task-matched trajectory paths while ensuring path feasibility, verifying the good adaptability and robustness of this algorithm for complex unstructured environments under multi-task driving conditions.

## 1. Introduction

In recent years, with the development of machine learning and autonomous driving technology, the application scenarios of unmanned equipment have become increasingly widespread. As a typical type of unmanned equipment, UGVs are also widely used in disaster relief, security patrols, mining transportation, logistics distribution, and other fields and have high research value. However, in some complex environments, how to enable a UGV to efficiently and safely avoid obstacles and dynamically change path-planning strategies according to the characteristics of the current task has also become one of the key technical challenges in the research of intelligent unmanned systems.

Currently, path-planning algorithms are mainly divided into three categories: sampling-based methods, trajectory-generation-based methods, and graph-search-based methods. Among these are sampling-based methods such as the Probabilistic Roadmap (PRM) and Rapidly Exploring Random Tree (RRT) algorithms. The PRM algorithm constructs a global graph through node sampling and connectivity detection within a predefined space and is suitable for global path searching in static environments [1,2]; The RRT algorithm has the advantage of fast searching by continuously expanding from the starting point to the target area, making it suitable for dynamic environments. However, the optimality and smoothness of the generated path cannot be guaranteed [3,4,5,6,7].

In contrast, path-planning methods based on a graph search, such as the Dijkstra algorithm and the A* algorithm, are still widely used in complex environments due to their clear structure and controllable search process. The A* algorithm combines heuristic search strategies with cost function and has become the core algorithm in many practical path-planning systems [8]. However, when constructing paths in grid maps, the traditional A* algorithm often results in paths composed of a large number of adjacent grid nodes, leading to issues such as excessive node expansion, redundant paths, and low search efficiency. This is particularly challenging in scenarios with significant terrain variations or dense threat zones [9,10,11,12,13,14].

To address this problem, Duan, C. proposed an evaluation function to optimize the hybrid A* algorithm, which makes the paths obtained by the algorithm smoother by introducing angular penalty coefficients, in addition to successfully improving the search efficiency of the optimal paths by introducing a node-expansion method for the optimal step size [15]. Sheng W proposed an automated parking trajectory-planning method for unstructured environments in narrow aisles, where the global path is searched and planned by a multilevel hybrid A* algorithm, and then the rough path is optimized by a numerical optimization layer with a time-optimized velocity profile to generate an accurate trajectory [16]. Meng T proposed an improved hybrid A* algorithm for the unfamiliar and complex scenarios of automated parking, integrating field potentials in the path-search phase to avoid obstacles and adopting a multi-stage dynamic optimization strategy, which divides the path planning into multiple phases, to effectively improve the path safety [17]. Chi Z proposed a non-omni-directionally constrained robot path-planning method and designed an improved hybrid A* algorithm to achieve high-precision global path planning and navigation. The algorithm combines the model predictive control (MPC) theory for local path planning and obstacle avoidance, which effectively improves the path-planning efficiency and navigational accuracy in complex environments [18]. Zhao Y proposed a hybrid A* path-planning algorithm based on multi-objective constraints. The method introduces a penalty mechanism for obstacles based on the traditional hybrid A* algorithm to avoid the path from getting too close to the obstacles while considering the smoothness and safety of the path through multi-objective optimization. In the path-fitting stage, a post-processing method is used to generate a drivable path with a continuously varying curvature using multi-objective constraints [19]. Zhao K proposed an improved hybrid A* path-planning algorithm for automated parking scenarios for cars, which searches for a path to reach the target point by using the circular spline curve as a reference path and the points on the reference path as the target point of the hybrid A* algorithm [20]. Sun Y proposed a hybrid path-planning method combining an improved A* algorithm with the Dynamic Window Approach (DWA), where a bidirectional search strategy and an adaptive heuristic function are introduced into global path planning, which effectively improves the search efficiency, reduces redundant search nodes, and improves the path smoothness by using cubic B-spline curves [21].

Although hybrid A* has produced many results in optimizing node search efficiency and path smoothness, there is still room for further research to verify its effectiveness in complex environments. Ren B proposed variable radius RS curves to improve the flexibility of the path search, while path optimization based on Bessel curves and the gradient-descent method was used to improve the path quality [22]. Chi X optimized the node expansion direction by increasing the resolution of the steering angle and combining a reverse search strategy with the A* algorithm’s cost map, which allows for generating shorter and smoother paths in parking scenarios [23]. Deng H designed a trajectory tracker based on a vehicle dynamics model, which integrally plans the path trajectory under obstacle-avoidance conditions by introducing an obstacle-avoidance function [24].

Fuzzy logic control is widely used in path planning in complex unknown environments due to its advantages in handling uncertain information [25,26,27,28]. Traditional fuzzy controllers have certain limitations in rule formulation and parameter adjustment, which can be effectively solved by combining fuzzy control with neural networks to form fuzzy neural networks [29,30,31,32,33]. This is because the neural network can effectively deal with the uncertainty of environmental information and improve the robustness of path planning. Secondly, the adaptive learning capability of neural networks enables the system to adjust the parameters online and adapt to the dynamically changing environment [34,35,36,37,38,39,40]. Therefore, optimizing the cost weights in the path-planning process by means of fuzzy neural networks can make the generated paths smoother, improve the feasibility and safety of the paths, and increase the efficiency of the path search.

Based on a comprehensive analysis of the limitations of existing path-planning algorithms, how to effectively integrate terrain elevation information with unmanned vehicle motion models in complex terrain environments, improve path-planning accuracy and feasibility, reduce overall path costs, and achieve planning responses to hazardous areas have become key technical issues that need to be addressed urgently. In response to the above challenges, this paper proposes an Adaptive Dynamic Path (ADP) planning algorithm for unstructured three-dimensional terrain environments to improve the path feature rationality and environmental adaptability of ground unmanned vehicles in complex environments. The algorithm makes full use of three-dimensional maps containing elevation information and combines the dynamic characteristics of the chassis suspension of unmanned vehicles to construct an interference detection mechanism between the vehicle model and the terrain, thereby achieving precise modeling and dynamic constraints on the terrain passability. At the same time, multiple cost items (such as terrain roughness, obstacle distance, and danger-zone risk) are introduced into the path cost-evaluation process. By introducing a neural network and combining it with the Euclidean distance constraint mechanism, the weight coefficients in the path cost function are dynamically adjusted to construct a cost function that can be dynamically adjusted according to the task requirements.

## 2. A Costly Hybrid A* Algorithm Incorporating Fuzzy Neural Networks

This paper proposes a dynamic cost factor hybrid A* path-planning algorithm that integrates fuzzy neural networks. By constructing a corresponding set of performance indicators and using multiple key indicators, a path-evaluation function is constructed using a weighted aggregation method.

**Figure 1:**
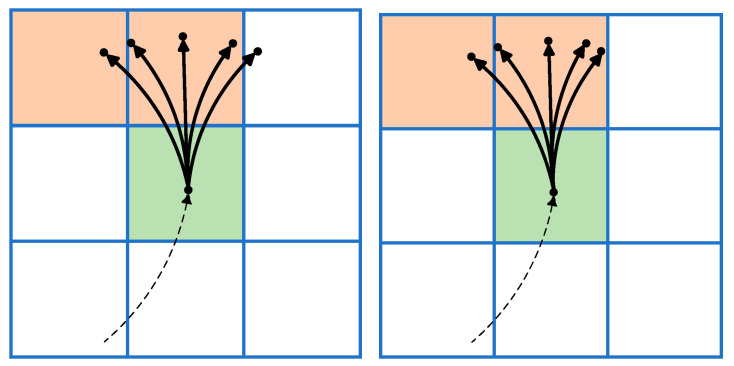
Features of the Hybrid A* Algorithm.

First, based on the degrees of freedom of the autonomous vehicle, its steering angle is discretized. Then, combining the forward and reverse gears, a set of alternative trajectories is formed that extends to neighboring areas. Finally, the alternative trajectories are evaluated to determine the optimal option, as shown in Figure 1. The green area indicates the current location, and the orange area indicates the drivable area.

Determine whether the unmanned vehicle will collide with obstacles by calculating the minimum distance between the grid on the planned path and the obstacles, as shown in the following formula:

(1) $$d(n_{i},o)=\underset{n_{i}\in o}{min}||n_{i}-o_{j}|{|}_{2}$$

where $n_{i}$ is the grid point on the path, O is the set of obstacles, and a collision is considered to have occurred if there exists $d(n_{i},O)$ that is less than the safe-distance threshold.

When there are no obstacles within the pre-planned path, the optimal path from the starting point to the goal point is output. Save the steering angle with the forward direction as shown in the following equation:

(2) $$p_{o}=\mathrm{argmin}\sum_{i=1}^{m-1}\left||n_{i+1}-n_{i}|\right|$$

(3) $$\theta_{i}=arctan(\frac{y_{i+1}-y_{i}}{x_{i+1}-x_{i}})$$

where $p_{o}$ is the optimal path, m is the total number of nodes on the path, $n_{i}$ indicates the i path node on the path, and $\theta_{i}$ is the steering angle from node $n_{i}$ to $n_{i+1}$.

Unlike traditional hybrid A* path-planning serial algorithms, this algorithm treats the pitch angle, roll angle, and ground clearance of the UGVs at discrete nodes on the map as passable features of the three-dimensional terrain. By combining the current road environment with the task objectives of the unmanned vehicle, fuzzy neural networks are used to train the weight coefficients of various costs under different conditions. Finally, an evaluation function is used to obtain the optimal path.

### 2.1. Functional Framework of Fuzzy Neural Networks

First, collect external environmental information from the map and obtain the current vehicle motion status. Then, input the constraints from the current path-planning process into the fuzzy neural network to obtain the dynamic cost weighting coefficient for the optimal path evaluation index, thereby calculating the best path.

By inputting environmental status perception quantities, such as the type of task for the unmanned vehicle, obstacle density, ground unevenness, energy margin, and vehicle health status, the data in the input layer is normalized and input into the second layer. The input–output relationship of the nodes in this layer is as follows:

(4) $$x_{i}^{\left(1\right)}=x_{i}^{\left(0\right)}\;i=1,2,3,4,5$$

where $x_{i}^{\left(0\right)}$ is the original output layer, and $x_{i}^{\left(1\right)}$ is the first-layer input value.

The second layer of fuzzification converts the input environmental state perception parameters into fuzzy variables through corresponding membership functions., where $x_{1}$ is a preset value including four mission modes: conveying, reconnaissance, sweeping, and UGV take-off and landing. $x_{2}$ can be fuzzed as $k_{2}$: no barriers, no access, and occupancy in general. $x_{3}$ can be blurred into $k_{3}$: completely flat and undulating. $x_{4}$ can be blurred into $k_{4}$: Lack of energy and sufficient energy. $x_{5}$ can be fuzzified as $k_{5}$: vehicle failure and vehicle health. The fuzzified parameters are input into the fuzzy inference layer.

The third layer is the fuzzy inference layer, which is defined in this paper as the sensitivity of each input parameter to different output costs. For example, in conveying mode, fuzzy reasoning schemes include high sensitivity to terrain uncertainty, prioritizing avoidance of obstacles, sensitivity to gear changes and steering changes, prioritizing avoidance of danger points, and sensitivity to road bumpiness.

The fourth layer is the normalization layer, with the same number of nodes as the third layer, and it implements the normalization computation as follows:

(5) $$x_{j}^{\left(4\right)}=\frac{x_{j}^{\left(3\right)}}{\sum_{i=1}^{324}x_{i}^{\left(3\right)}}\;j=1,2,3,\ldots n$$

where $x_{j}^{\left(3\right)}$ is the output of the j node, and $x_{j}^{\left(4\right)}$ is the normalized result.

The fifth layer is the output layer that converts the inference results into specific cost weights and passes them to the path-planning module as shown in the following equation:

(6) $$y_{i}=\sum_{j=1}^{324}w_{ij}x_{j}^{\left(4\right)}\;i=1,2,3,\ldots 8;\;j=1,2,3,\ldots n$$

where $y_{i}$ corresponds to the weighting coefficients of the eight cost factors in path planning, including the terrain uncertainty cost, obstacle distance cost, road length cost, gear shift cost, cornering cost, steering change cost, hazardous point distance cost, and bumps cost.

By dividing the four input parameters into low, medium, and high language values, the weighted sum of each cost weight can be obtained as

(7) $$J_{total}=\sum_{i=1}^{8}w_{i}f_{i}$$

where $w_{i}$ is the weight coefficient of item i and $f_{i}$ is the path cost component of item i.

Different input parameters affect the output cost weighting strategies as shown below, and ten typical cases of them are listed, as shown in Table 1. In the learning process of a fuzzy neural network, the error function is used to measure the deviation between the network output and the desired output, as shown in the following equation:

(8) $$E=\frac{1}{2}\sum_{i=2}^{r}(y_{di}-y_{i}{)}^{2}$$

where E is the error cost function (the smaller its value, the closer the network output is to the desired value), $y_{di}$ is the desired output of the i-th sample, and $y_{i}$ is the actual output calculated by the fuzzy neural network.

**Figure 2:**
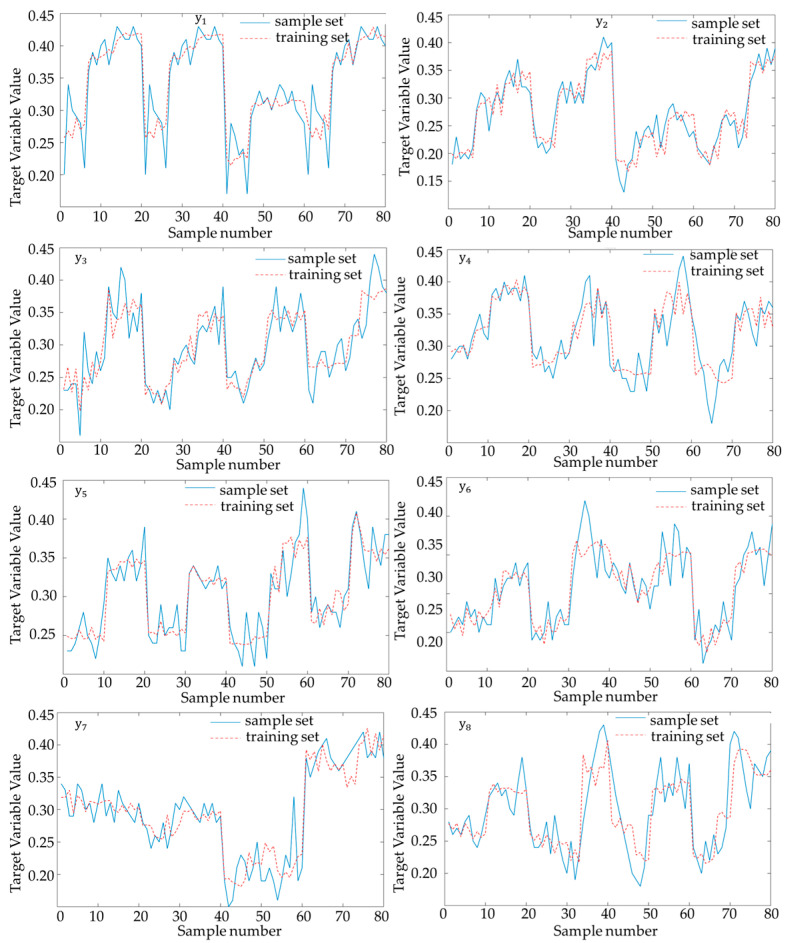
Neural network training results.

In order to reduce the error cost function, updates are performed by gradient descent so that the network is constantly adapted to different path-planning scenarios, as shown in the following equation:

(9) $$w_{ij}(k+1)=w_{ij}(k)-\eta \frac{\partial E}{\partial w_{ij}(k)}$$

(10) $$c_{ij}(k+1)=c_{ij}(k)-\eta \frac{\partial E}{\partial c_{ij}(k)}\;$$

(11) $$\sigma_{ij}(k+1)=\sigma_{ij}(k)-\eta \frac{\partial E}{\partial \sigma_{ij}(k)}$$

where k is denotes the current number of iterations and η is the learning rate, which is set between 0–1 in this chapter. $w_{ij}$ is the gradient descent update formula for neural network weights, $c_{ij}$ is the membership function, and $\sigma_{ij}$ is the membership function width; by constantly adjusting the size of $w_{ij}$, $c_{ij}$ and $\sigma_{ij},$ the path can be made to adjust dynamically with the environment. The training results are shown in Figure 2.

As can be seen in Figure 2, the predicted cost coefficients learned in the training set are able to fit the cost output weights given in the sample set well, reflecting good prediction performance.

**Figure 3:**
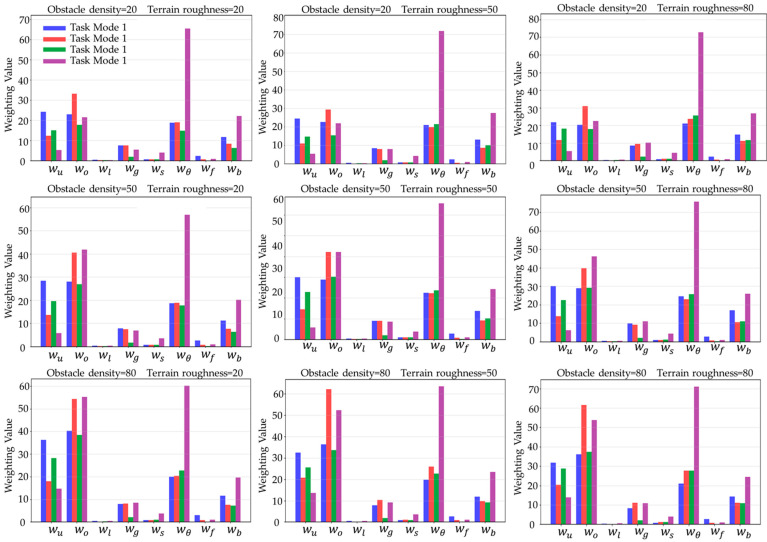
Cost weighting values in different environments.

**Figure 4:**
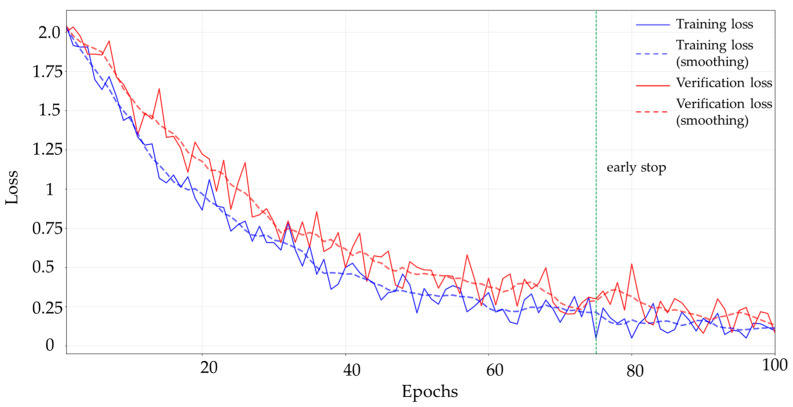
Training loss and validation loss curves.

To verify the adaptability of the neural network model in different environments, nine sets of combined scenarios were designed under different environmental conditions, corresponding to combinations of obstacle density (20, 50, 80) and ground roughness (20, 50, 80). In each set of scenarios, the eight-dimensional cost factor weight values of the neural network output were calculated for four task modes, as shown in Figure 3:

Figure 4 represents the loss curves of the training and validation sets, which shows that the training loss gradually decreases and tends to converge with the increase in the number of iterations, while the validation loss remains stable, indicating that the model is able to obtain a better generalization ability under different task modes. The fuzzy rules for different terrain environments are shown in Table 1.

### 2.2. Path-Planning Algorithm Flow

In this paper, the algorithm adds the kinematic constraints of the vehicle with respect to the traditional A* algorithm, i.e., the input data is extended to a 3-dimensional space [x,y,θ], where x,y are the 2D coordinates of the vehicle, and θ is the heading angle of the unmanned vehicle, which allows for the continuous planning of the unmanned vehicle’s position and orientation in a discretized grid [41].

The pseudocode for this article is given in Algorithm 1.
**Algorithm 1** ADP Path-Planning Algorithm**Input:** $n_{start}$$(s_{x},s_{y},\theta_{s}$$),\;n_{goal}$$(g_{x},g_{y},\theta_{g}$$),\;d_{obs}$$\;(o_{x},o_{y},o_{z}$$),\;{Land}_{3d}$, uncertainty map unc, taskmode  $(T_{1}-T_{4}$)**Output:** path $P_{t}$ **BEGIN:** 1: Initialize: 2: reso ← resolution; step ← reso × 8 3: W ← Normalize(MLP(Fuzzify(TaskMode))) // task-aware cost weights 4:$\;\mathrm{KD}\;\leftarrow \;\mathrm{KDTree}(o_{x},o_{y},o_{z}$) 5:$\;h_{map}$ ← A ×$\;(g_{x},g_{y}$) 6: $\;d_{obs}$(x,y) ← dist to nearest obstacle 7:$\;\mathrm{Open}\;\leftarrow \;\{n_{start}$}; Closed ← ∅ 8: Q ← PriorityQueue(f = g +$\;W.H_{c}$· h) 9:     while Open ≠ ∅: 10:      $\;n_{c}$$\;\leftarrow \;\mathrm{argmin}\;Q;\;\mathrm{Open}\;\leftarrow \;\mathrm{Open}\backslash \{n_{c}$$\};\;\mathrm{Close}\;\leftarrow \;\mathrm{Closs}\;\cup \;\{n_{c}$} 11:        $\;\mathrm{if}\;\|n_{c}$$-n_{goal}$${\|}_{2}\;<\;{RS}_{thresh}$: 12:          $\;\mathrm{RS}\;\leftarrow \;\mathrm{CalcAllRS}(n_{c}$$,\;n_{goal}$) 13:              for P ∈ cost(RS): 14:                 if NoCollision(P): 15:                 $\;\mathrm{return}\;\mathrm{Reconstruct}(\{n_{c}$) + P 16:       for: 17:          x$\;\leftarrow \;x_{c}$; y$\;\leftarrow \;y_{c}$; θ$\;\leftarrow \;\theta_{c}$ 18:         $\;\mathrm{valid}\leftarrow \mathrm{True};\;\varphi_{0}$$,\;\rho_{0}$ ← ∇H(x,y) 19:            for 20:              x ← x + d · reso · cos(θ); y ← y + d·reso·sin(θ) 21:              θ ← θ + d·reso·tan(δ)/L 22:                if (x,y) ∉ bounds ∨ KD.collide(x,y): valid ← False; break 23:                  φ, ρ ← ∇H(x,y) 24:                    if |φ$|\;>\;\varphi_{max}$ ∨ |ρ$|>\rho_{max}$$\;\vee \;(H_{max}$$\;-\;H_{min}$$)\;>\;h_{max}$:valid 25:                       False; break 26:                         if valid: continue 27:                    f ← compute_cost(x, y,  θ, δ$,\;W,\;{Land}_{3d}$$,\;\mathrm{map}\;\mathrm{unc},\;d_{obs}$, fire, bump, steer) 28:          node ← (x, y,  θ, g′, f′) 29:             if node ∉ Closed: 30:              $\;\mathrm{if}\;\|(x,y)\;-\;(g_{x}$$,g_{y}$$){\|}_{2}\;<\;\varepsilon$ ∧ |θ$\;-\;\theta_{g}$| < ε: 31:                $\;P_{t}$ ← Reconstruct(node) 32:                 $\;\mathrm{return}\;P_{t}$ **End**

By discretizing the coordinates of the starting point and the target point and normalizing the angle to the range of ±π, the distance from each node to the target point, the shortest distance to the obstacle, and the steering angle and driving direction during the movement are recorded during the search process. The mixed cost value is calculated by superimposing the current node cost and the heuristic cost, and the expansion order is determined based on the distance between the current position of the unmanned vehicle and the target point. Take the target point as the starting point for backward expansion, gradually calculate the minimum cost to each node, each time selecting the node with the lowest cost from the open set. Retrieve all its possible ways of movement, generate a new node, and calculate the cost, and when the new node is not visited or there exists a better path, update its cost and add it to the open set and the priority queue, and, ultimately, form a heuristic cost map that contains all the nodes that have been visited. The specific steps are as follows:

(1) Input the target node n, the coordinates of the obstacle E(x,y), the resolution reso, and the turning radius rr to obtain the inspired map and the distance from the UGV to the obstacle.
(2) The target node is discretized, and the discretization formula can be expressed as
  (12) −E(x,y)=E(x,y) / reso
(3) Call the map parameters and output the information, such as the obstacle grid and map boundary; initialize the open and closed sets and add the target node to the open set; the closed set is set to empty. Also, initialize the priority queue and add the target node to the queue.
(4) When a node exists in the open set, the node with the smallest cost is taken out and added to the closed set and removed from the open set. Subsequently, for each direction of motion, generate new nodes and calculate their coordinates, cost, and parent-node index. If the node is out of bounds or within an obstacle, it is skipped. Calculate the index of the node, and if it is not in the closing set, then consider the following two cases: 1. If it is already in the open set and the new cost is better, then update the cost with the parent-node index. 2. If it is not in the open set, then add the node to the open set and put it in the priority queue.
(5) Initialize the cost map, traverse the closure set, and fill the final cost of each node into the corresponding position in the cost map.
(6) Return the heuristic cost map and obstacle distance mapping.

Local dynamic obstacle avoidance is achieved through fuzzy neural network control combined with Euclidean distance constraints. A cost weight adaptation method based on fuzzy neural networks is used to dynamically adjust the weights of various costs based on factors such as obstacle density, vehicle stability requirements, and the current operating status of the unmanned vehicle. By inputting multi-dimensional environmental parameters such as obstacle distribution, path curvature, and speed limits and combining them with fuzzy rules for inference, the system outputs dynamically adjusted weights in real time.

## 3. Multi-Factor Conditional Weighting Function Model

In the path-planning process, the element evaluation function directly affects the robustness of the path-planning results. This paper constructs a multielement cost model f to obtain the optimal path, as shown in the following equation:

(13) $$f=w_{u}f_{u}+w_{o}f_{o}+w_{l}f_{l}+w_{g}f_{g}+w_{s}f_{s}+w_{\theta }f_{\theta }+w_{f}f_{f}+w_{b}f_{b}$$

where f is an indicator of the integrated evaluation function; $f_{u}$, $f_{o}$, $f_{l}$, $f_{g}$, $f_{s}$, $f_{\theta }$, $f_{f}$, and $f_{b}$ are the terrain uncertainty cost, obstacle distance cost, road length cost, gear shift cost, cornering cost, steering change cost, hazardous point distance cost, and bumps cost; and $w_{u}$, $w_{o}$, $w_{l}$, $w_{g}$, $w_{s}$, $w_{\theta }$, $w_{f}$, and $w_{b}$ are their corresponding weights.

The terrain uncertainty cost $f_{u}$ is derived from the environmental sensing system, which rasterizes the ground and assigns values to each raster to represent the uncertainty risk.

With regard to the obstacle distance cost $f_{o}$, here, each trajectory line is expanded into an arc band by combining the outer dimensions of the unmanned vehicle. To facilitate the calculation of the path-planning algorithm, the arc band is gridded, and the continuous path is represented as a series of discrete nodes, as shown in Equation (14):

(14) E(x,y)=h(x,y)

Through calculation (1), the minimum distance between the grid on the planned path and the obstacle is calculated to determine whether there is a collision risk for the unmanned vehicle. If d is less than the safety distance threshold $l_{a}$, a collision is deemed to have occurred. When the minimum distance of the proposed trajectory point from the static obstacle is less than the parameter $l_{b}$, the risk level is *A*. Beyond that, the risk decreases nonlinearly and rapidly. This is expressed in Equation (15):

(15) $$f_{o}=k\times \left\{\begin{array}{c} \;\;\;\;\;A\;\;\;\;\;\;\;\;\;\;\;\;\;\;\;\;\;\;\;\;\;\;\;\;\;\;\;\;dis<l_{b}\\ \frac{1}{(dis-l_{b}{)}^{3}+1/A}\;\;\;\;\;\;\;\;\;\;\;\;\;dis\geq l_{b} \end{array}\right.$$

The road length cost $f_{l}$ corresponds to the length of the trajectory; the shift cost $f_{g}$ is the switch between forward and reverse, which will bring about longitudinal vibration as the longitudinal speed of the unmanned vehicle alternates. The cornering cost $f_{s}$ corresponds to the deflection angle of the steering wheel of the unmanned vehicle, which poses a risk to the stability of the unmanned vehicle’s driving and has a strong correlation with the vehicle speed, which is not conducive to high-speed driving of the unmanned vehicle. The steering change cost $f_{\theta }$ responds to the effect of the amount of steering-wheel deflection angle change on the trajectory continuity of an unmanned vehicle.

The hazardous point distance cost $f_{f}$ reflects some of the forms of hazards encountered by unmanned vehicles, and the hazard distance cost is affected by the distance parameter between the intensity of the hazard and the protection capability of unmanned vehicles, as shown in the following equation. When the minimum distance of the proposed trajectory point from the danger point is less than the $l_{kill}$ parameter, it means that the risk level is infinite, and exceeding $l_{kill}$ means that the risk decreases.

(16) $$f_{f}=k\times \left\{\begin{array}{c} \;\;\;\;\inf\;\;\;\;\;\;\;\;\;\;\;\;dis<l_{kill}\\ \frac{1}{dis-l_{kill}+1/F}\;\;\;\;\;\;\;dis\geq l_{kill} \end{array}\right.$$

**Figure 5:**
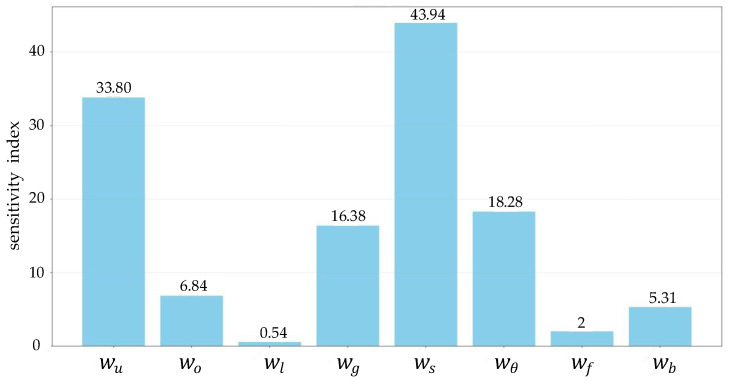
Sensitivity of path-planning results to eight different costs.

The chassis bump cost $f_{b}$ can be characterized by detecting the degree of front/rear “lift/lowering” of the vehicle over short distances and changes in the lateral camber, as shown in the following Equation (17):

(17) $$f_{b}=\sigma \times (|\Delta \theta_{pitch}|+|\Delta \theta_{roll}|)$$

where $\Delta \theta_{pitch}$ is the change in pitch angle, $\Delta \theta_{roll}$ is the change in the roll angle, and σ is the coefficient of bumping costs, controlling the weight of bumping costs in the total costs. For each cost factor, calculate its coefficient of variation (CV), which is the standard deviation divided by the mean. By calculating the proportion of each cost factor in the total cost, the sensitivity index for different costs can be obtained, as shown in Figure 5.

### 3.1. Chassis Interference Analysis

**Figure 6:**
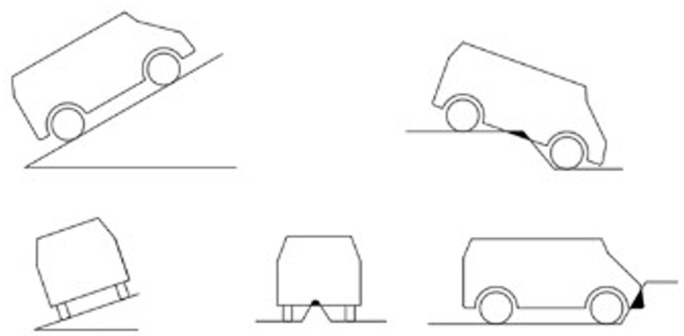
Unmanned vehicle passage restraint schematic.

For the case of interference with the ground, this paper considers several scenarios that may have occurred, as shown in Figure 6.

**Figure 7:**
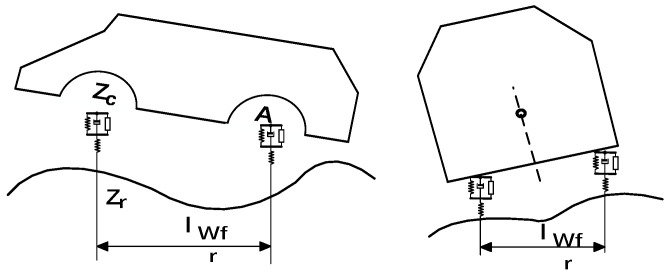
Unmanned vehicle modeling on undulating roads.

The passability is expressed in terms of pitch-angle constraints and side-camber constraints, and the localized height of the ground under the vehicle cannot exceed the minimum ground-clearance constraint of the chassis. When a wheeled unmanned vehicle with independent suspension is traveling at low speed on an uneven road surface, the chassis needs to be checked for interference with ground bumps; for this purpose, it is necessary to obtain information about the interference of the underbody plane, and if there is an interference, this trajectory will not be selected. Since the body is supported by four suspensions, the exact position of the underbody plane can be deduced by calculating the coordinates of the connection points between the suspensions and the body. The independent-suspension-wheeled unmanned vehicle model is shown in Figure 7.

By reducing the suspension to springs and dampers in parallel, in series with the tires, the tires are reduced to springs. The tires are further simplified to rigidity, since they are much more elastic than the suspension. In the study of the passing ability of unmanned vehicles, the role of shock absorbers is neglected, and only the steady-state case is considered, since the main focus is on the low-speed driving condition.

The spatial position of the suspension and the body is modeled and solved by setting the elevations of the four wheels in contact with the ground to be $z_{rfl}$, $z_{rfr}$, $z_{rrl}$, and $z_{rrr}$; the elevations of the upper connection points of the suspension and the body to be $z_{cfl}$, $z_{cfr}$, $z_{crl}$, and $z_{crr}$; the vertical forces of the suspension and the body to be $P_{fl}$, $P_{fr}$, $P_{rl}$, and $P_{rr}$, which correspond to the left front wheel, the right front wheel, the left rear wheel, and the right rear wheel; wheelbase to be $l_{wlr}$, and the axle base to be $l_{wfr}$. The four suspension springs have the same stiffness k, and the initial length of the spring is l. The elevation of the point where the wheels touch the ground can be obtained by querying the 3D map based on the XY-projected coordinates of the unmanned vehicle on the ground. Then the compression value of the spring force is

(18) $$\left\{\begin{array}{c} z_{cfl}=z_{rfl}+l-p_{fl}/k\\ z_{cfr}=z_{rfr}+l-p_{fr}/k\\ z_{crl}=z_{rrl}+l-p_{rl}/k\\ z_{crr}=z_{rrr}+l-p_{rr}/k \end{array}\right.$$

The body pitch and side lean angles are

(19) $$\theta_{pitch}=\frac{\left(\arctan\frac{z_{cfl}-z_{crl}}{l_{wfr}}+\arctan\frac{z_{cfr}-z_{crr}}{l_{wfr}}\right)}{2}$$

(20) $$\theta_{roll}=\frac{\left(\arctan\frac{z_{cfr}-z_{cfl}}{l_{wfr}}+\arctan\frac{z_{crr}-z_{crl}}{l_{wfr}}\right)}{2}$$

Equations (18)–(20) then simplify to

(21) $$\theta_{pitch}=\frac{z_{cfl}-z_{crl}+z_{cfr}-z_{crr}}{2*l_{wfr}}=\frac{k(z_{rfl}-z_{rrl}+z_{rfr}-z_{rrr})+P_{rl}+P_{rr}-P_{fl}-P_{fr}}{2*l_{wfr}*k}$$

(22) $$\theta_{roll}=\frac{z_{cfr}-z_{cfl}+z_{crr}-z_{crl}}{2*l_{wlr}}=\frac{k(z_{rfr}+z_{rrr}-z_{rrl}-z_{rfl})+P_{fl}+P_{rl}-P_{fr}-P_{rr}}{2*l_{wlr}*k}$$

Assuming that the center of mass of the unmanned vehicle is coincident with the geometric center in the XY plane, this can be obtained based on the principle of the equilibrium of longitudinal and transverse torques of the body:

(23) $$\left(P_{fl}+P_{rl}\right)\left(l_{wlr}-z_{m}\sin\theta_{roll}\right)=\left(P_{fr}+P_{rr}\right)\left(l_{wlr}+z_{m}\sin\theta_{roll}\right)$$

(24) $$\left(P_{fl}+P_{fr}\right)\left(l_{wfr}+z_{m}\sin\theta_{pitch}\right)=\left(P_{rl}+P_{rr}\right)\left(l_{wfr}-z_{m}\sin\theta_{pitch}\right)$$

where $z_{m}$ is the height of the center of inclination from the center of mass. By the principle of balance of forces in the vertical direction,

(25) $$P_{fl}+P_{fr}+P_{rl}+P_{rr}=mg$$

where m is the unmanned vehicle spring mass. Since the four suspensions are in a plane with the upper connection point of the body and do not change this feature with body bump pitch, sideways tilt, and rotation, the following can be obtained:

(26) $$z_{cfl}+z_{crr}=z_{cfr}+z_{crl}$$

Bringing in Equation (26) produces

(27) $$k(z_{rfl}+z_{rrr}-z_{rfr}-z_{rrl})=P_{fl}+P_{rr}-P_{fr}-P_{rl}$$

The system of Equations (23)–(26) can be obtained by combining the following equations:

(28) $$\left\{\begin{array}{l} \begin{array}{l} P_{fl}+P_{fr}+P_{rl}+P_{rr}=mg\\ k(z_{rfl}+z_{rrr}-z_{rfr}-z_{rrl})=P_{fl}+P_{rr}-P_{fr}-P_{rl} \end{array}\\ \begin{array}{l} \left(P_{fl}+P_{rl}\right)\left(l_{wlr}-z_{m}\sin\theta_{roll}\right)=\left(P_{fr}+P_{rr}\right)\left(l_{wlr}+z_{m}\sin\theta_{roll}\right)\\ \left(P_{fl}+P_{fr}\right)\left(l_{wfr}+z_{m}\sin\theta_{pitch}\right)=\left(P_{rl}+P_{rr}\right)\left(l_{wfr}-z_{m}\sin\theta_{pitch}\right) \end{array} \end{array}\right.$$

**Figure 8:**
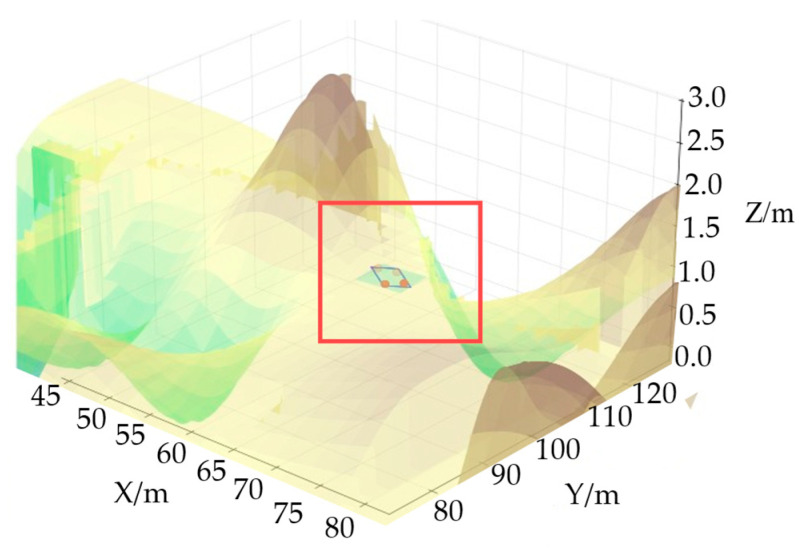
Chassis-road interference detection model diagram.

**Figure 9:**
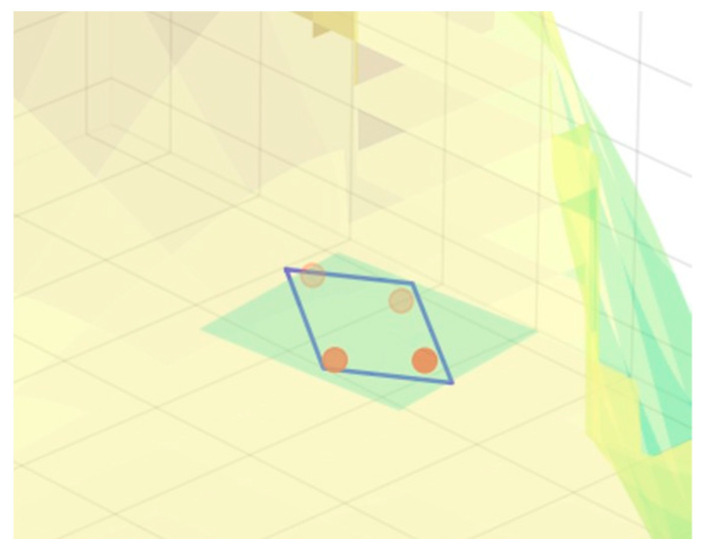
Localized enlargement of the chassis and road interference detection model.

The combination of the first two equations in the system of equations can simplify the four variables into two variables, and then the latter two equations are simplified by trigonometric functions, which are brought into Equations (21) and (22) to solve for the four pendant forces $P_{fl}$, $P_{fr}$, $P_{rl}$, and $P_{rr},$ which, in turn, yield the coordinates of the four upper connection points of the suspension to the body, as shown in Figure 8 and Figure 9.

As shown in Figure 9, compare the elevation of the lower part of the vehicle body with the plane to determine whether it will be higher than the plane. The blue box in the figure is a simplified outline of the unmanned vehicle, and the light blue rectangle below is the detection range for interference between the unmanned vehicle chassis and the ground. The four circular areas are interference detection points, with darker colors indicating stronger interference, thereby verifying whether there is a risk of “bottom scraping”.

### 3.2. Collision Detection Model

**Figure 10:**
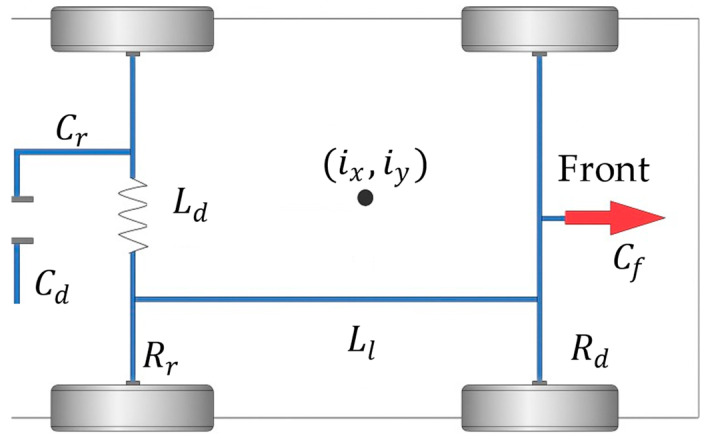
Sketch of unmanned vehicle model.

A sketch of the unmanned vehicle model in this paper is shown in Figure 10.

Take the center of the vehicle as the center of the circle, establish a circular collision detection area with a radius of half of the longitudinal projection of the vehicle plus a safety extension, and set the range of the radius of the collision detection as r. The calculation formula is as follows:

(29) $$r=\frac{L_{l}}{2}+\max(C_{f},C_{r})+d$$

where $L_{l}$ is the front and rear wheelbase, $C_{f}$ is the distance from the front axle to the front end of the vehicle, $C_{r}$ is the distance from the rear axle to the rear end of the vehicle, and d is the safety extension radius. Then the center coordinates $\left(c_{x},c_{y}\right)$ of the vehicle are

(30) $$c_{x}=i_{x}+\frac{L_{l}}{2}\cdot \cos(i_{x}\theta )$$

(31) $$c_{y}=i_{y}+\frac{L_{l}}{2}\cdot \sin(i_{y}\theta )$$

where $i_{x}$ and $i_{y}$ denote the rear-axle center coordinates of the current position of the vehicle, and $i_{y}\theta$ is the current heading angle of the vehicle. Convert the obstacle position $o_{x}$, $o_{y}$ from a global coordinate system to a vehicle local coordinate system with the center of the vehicle as the origin and the forward direction of the vehicle as the x-axis. The conversion is as follows:

(32) $$\left[\begin{array}{c} d_{x}\\ d_{y} \end{array}\right]=\left[\begin{array}{c} \cos(i_{x}\theta )\;\sin(i_{x}\theta )\\ -\sin(i_{y}\theta )\;\cos(i_{y}\theta ) \end{array}\right]\cdot \left[\begin{array}{c} o_{x}-c_{x}\\ o_{y}-c_{y} \end{array}\right]$$

where $d_{x}$ and $d_{y}$ denote the horizontal and vertical distances of the obstacle in the vehicle coordinate system, respectively; then, the collision detection equation of the vehicle is

(33) $$Col=\left|d_{x}\right|<r\cap \left|d_{y}\right|<\frac{C_{d}}{2}+d$$

If the absolute values of $d_{x}$ and $d_{y}$ are less than the vehicle’s collision detection radius and half of the vehicle’s width plus the extended safety range, respectively, the vehicle is considered to have collided with an obstacle. A collision occurs when the obstacle is within a rectangular area of length 2r and width $C_{d}+2d$ in the vehicle’s local coordinate system.

## 4. Experimental Simulation

This paper sets the steering-wheel angle range of the autonomous vehicle to ±30°, discretized at 10° intervals, and considers both forward and reverse directions. This means that the autonomous vehicle needs to traverse 12 local trajectories and evaluate and select the local trajectory with the minimum cost. This paper sets a map of 100 m × 150 m, which is gridded in units of 0.5 m. It is assumed that planning a path requires evaluating 400 grid points, which means that at least 4800 local trajectories need to be verified. In other words, 4800 calculations of vehicle posture and chassis collision detection are required. The autonomous vehicle will periodically perceive the above environmental information and accelerate the calculation of all nodes using KDTREE.

In addition, based on the dynamic cost adjustment mechanism and three-dimensional terrain interference modeling method proposed in this paper, the following settings were made during the modeling process:

First, obstacles are modeled using regular rectangular models. During the path cost calculation, the distance between path points and obstacles is measured using the Euclidean distance.

Secondly, the vehicle chassis is modeled as a regular rectangular prism structure, and its suspension system is approximated using a simplified geometric compression model. This is used to assess changes in vehicle posture and terrain interference on three-dimensional undulating terrain, which facilitates the calculation of passability costs.

In terms of terrain, 3D terrain data is treated as static information during path planning, meaning that the terrain elevation does not change over time during the planning phase. This assumption is applicable to applications based on pre-built maps or environments with minimal dynamic changes.

In addition, during the initialization phase of path planning, the task mode is predetermined by the upper-level decision-making system, and the neural network outputs the corresponding cost weights based on the input task type and environmental features. Therefore, there is no need to dynamically switch task modes during the planning process.

**Figure 11:**
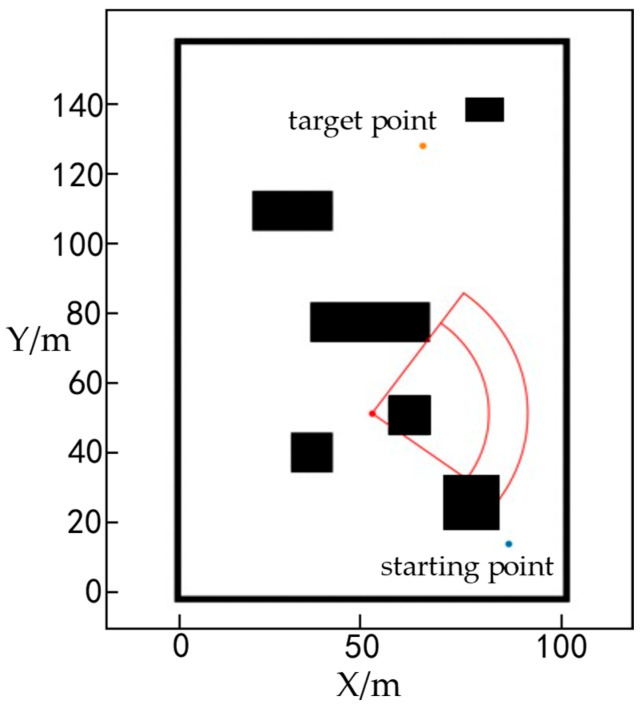
Simulation map for unmanned vehicle planning.

The above modeling assumptions are set to effectively control system complexity, highlighting the core advantages and practical effects of the algorithm in this paper in terms of task-driven dynamic weight adjustment and terrain-interference adaptive path planning. The final simulation environment map and path-planning results are shown in Figure 11. The four black squares in the figure represent obstacles, and the red sectors mark the hazard range, in which the outer area is the general hazard zone and the inner area is the lethal hazard zone. The orange node was randomly selected as the target location and the blue node as the starting location of the unmanned vehicle.

**Figure 12:**
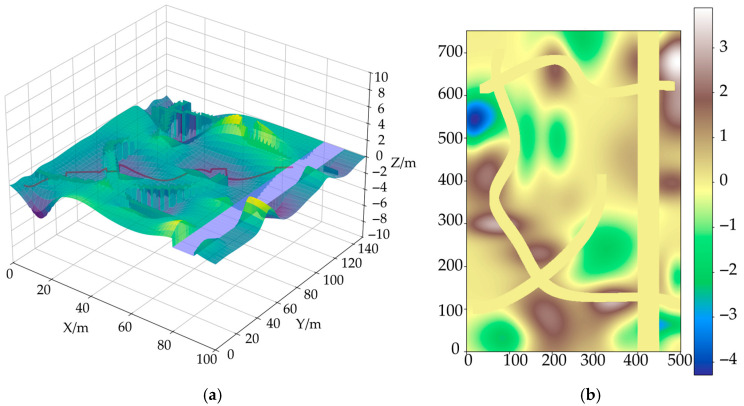
3D map model: (**a**) 3D map after introducing height information; (**b**) 3D path height map.

Figure 12a shows a three-dimensional map environment model that takes height information into account. To more intuitively reflect the height changes along the path, Figure 12b removes other environmental elements and retains only the height difference curve of the path.

**Figure 13:**
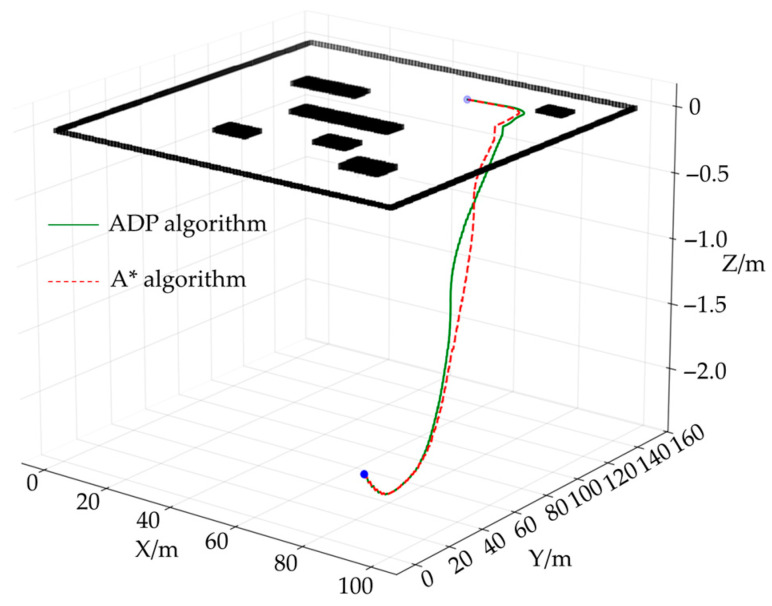
Three-dimensional comparison results of planned paths.

**Figure 14:**
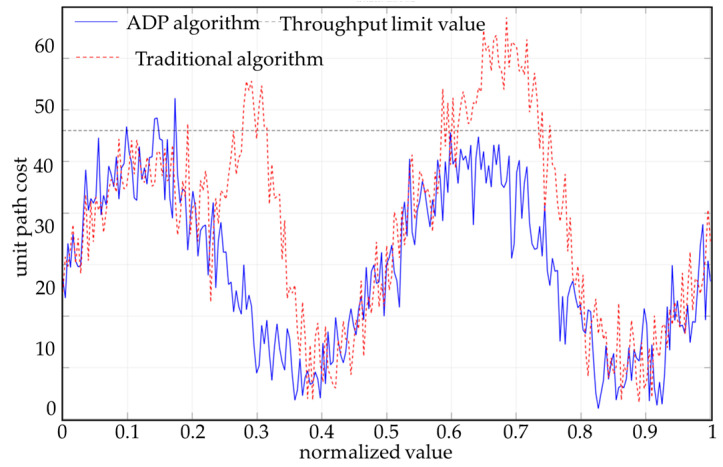
Chassis interference analysis results.

Figure 13 shows the difference between the ADP algorithm and traditional algorithms in terms of path cost distribution. The ADP algorithm comprehensively considers the impact of three-dimensional terrain on the chassis of unmanned vehicles during path generation, especially avoiding chassis interference and areas of severe bumpiness. Traditional algorithms are based only on two-dimensional terrain information and ignore chassis constraints, resulting in significant cost increases in some path segments (such as normalized positions x = 0.3 and x = 0.7). Compared with the traditional A* path-planning algorithm, which does not consider height information, the interference and pass rate are shown in Figure 14.

To further quantify the passability of a path, this paper introduces the path passability-rate metric, defined as the proportion of points in the path where the unit cost is below a certain safety threshold. This paper sets the threshold at 1.6. The calculation results for the pass rate are as follows: The path pass rate for the ADP algorithm is 98.4%, indicating that most path areas are safe for passage; the path pass rate for the traditional algorithm is 81.6%, with a significant number of potential interference or bumpy risk areas. The “path pass rate” refers to the proportion of paths in the entire route that vehicles can traverse smoothly without bottoming out or without excessive bumping.

**Figure 15:**
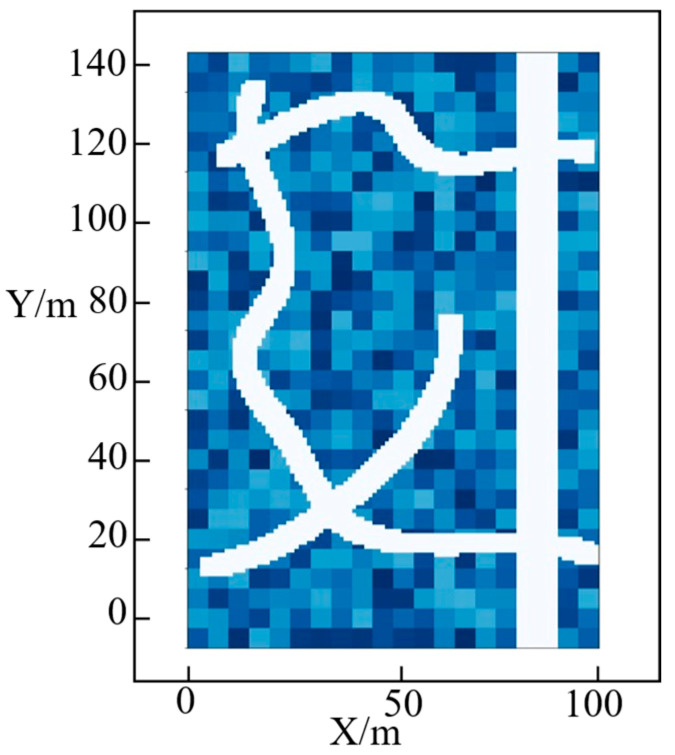
2D topographic map.

To facilitate observation of the experimental results, the three-dimensional terrain was mapped onto a two-dimensional plane using elevation projection, as shown in Figure 15. The white curves in the figure represent the four passable paths obtained from the three-dimensional map model in combination with the chassis suspension interference model of the unmanned vehicle.

**Figure 16:**
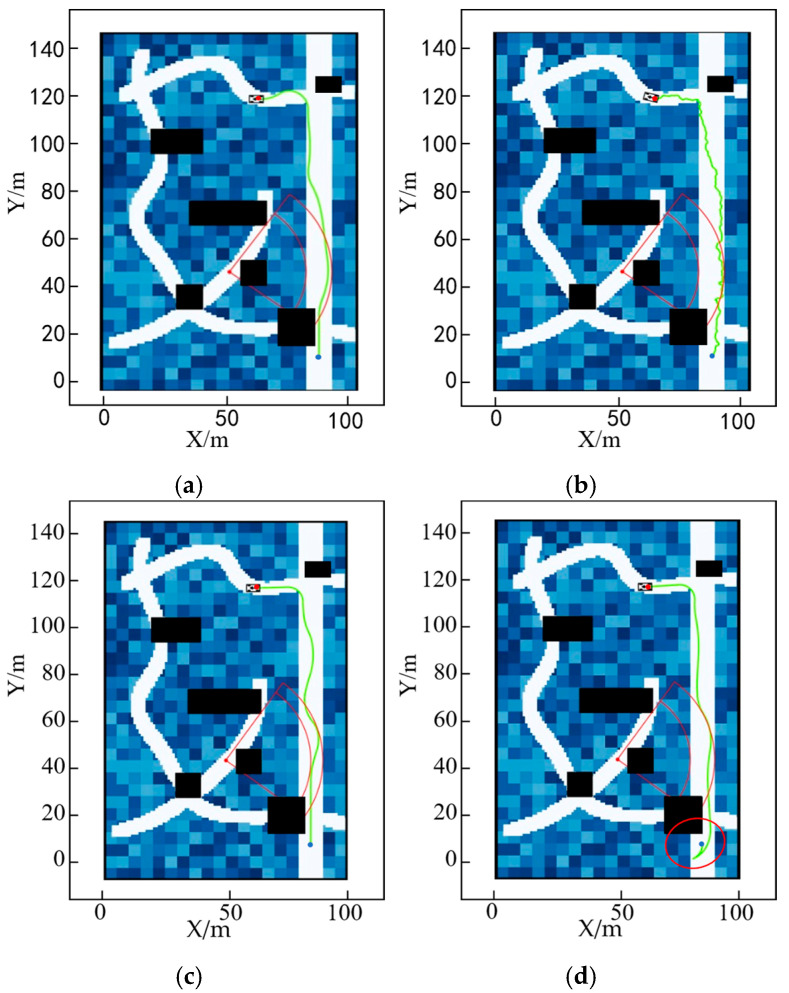
Comparison of planning results of different algorithms under low obstacle density: (**a**) A* algorithm; (**b**) FMT* algorithm; (**c**) Informed RRT* algorithm; (**d**) ADP algorithm.

In order to comprehensively evaluate the generality and adaptability of the ADP path-planning algorithm proposed in this paper, low-density obstacle scenes and high-density obstacle scenes were constructed separately. The former simulates open terrain or open roads and is mainly used to test the search efficiency and path smoothness of the algorithm; the latter simulates urban alleys or complex environments and is used to test the obstacle-avoidance ability and path smoothness of the algorithm in complex environments. First, the path planning results for a low-density obstacle scenario are shown in Figure 16.

The green curves in Figure 16 represent the path trajectories obtained by four different algorithms. As shown in Figure 16a,b, the traditional A* algorithm and FMT* algorithm only approximate the unmanned vehicle as a point mass and fail to consider collisions under the unmanned vehicle model, causing them to perform planning directly at the starting position, which leads to the risk of interference. The traditional A* algorithm performs the path search based on a heuristic search but does not fully consider dynamic terrain characteristics. Furthermore, when approaching the target location, it cannot effectively handle the vehicle’s steering angle, resulting in a large steering angle at the end of the planned path. The path-optimization effect of the FMT* algorithm is affected by the distribution of sampling points. When the sampling density is low, the path will be more tortuous, resulting in poor driving stability. When the sampling density is too high, although the path is more accurate, the computing efficiency is significantly reduced, affecting the real-time performance of the algorithm. Therefore, when the number of sampling points is the same as that of the traditional A* algorithm, FMT* has difficulty accurately capturing path details due to the sparse distribution of sampling points, resulting in paths with many small turns.

As shown in Figure 16c,d, the Informed RRT* algorithm is primarily based on distance-based heuristic optimization and does not integrate other environmental costs, resulting in longer detours around dangerous areas and multiple turns, thereby failing to fully utilize low-risk passageways. In this paper, when there are obstacles at the starting position, the ADP algorithm detects obstacles ahead based on the vehicle model data, performs collision detection, and then reverses to avoid the obstacles. After successfully bypassing the obstacles, the vehicle reaches the target position.

**Figure 17:**
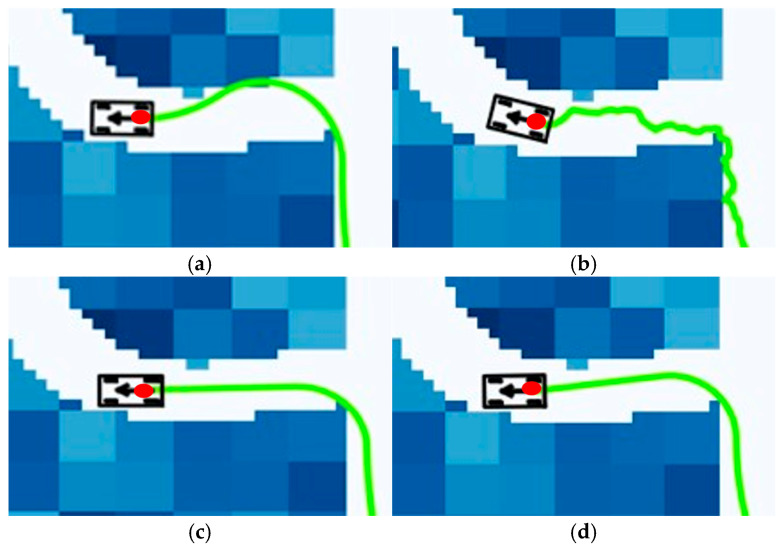
Local magnification of planning results for different algorithms under low obstacle density conditions: (**a**) A* algorithm; (**b**) FMT* algorithm; (**c**) Informed RRT* algorithm; (**d**) ADP algorithm.

As shown in the enlarged view in Figure 17 below, it can be observed that in the terminal path segment close to the target point, the green curves generated by the algorithm in this chapter and the Informed RRT* algorithm exhibit smoother turning characteristics, with the turning radius of the algorithm in this paper reduced by 37% compared with the traditional A* algorithm. Under the same number of sampling points, the FMT* algorithm exhibits a significantly higher number of minor turns at the end of the path compared with the A* algorithm and the algorithm presented in this chapter, failing to effectively control the adjustment of the unmanned vehicle’s body angle.

To quantify and compare the experimental results of the four algorithms, we conducted 20 sets of control experiments, comparing key indicators, including computation time, path smoothness (variance in curvature changes), and distance to the nearest obstacle. We calculated the variance and confidence interval for each set of data, with specific data shown in Table 2.

The path smoothness in the above table represents the variance in curvature changes, which reflects the magnitude of curvature changes between adjacent points. Although the algorithm described in this paper has a slightly higher computational time than traditional A* and FMT* algorithms, it has a smaller path smoothness and a larger nearest obstacle distance.

**Figure 18:**
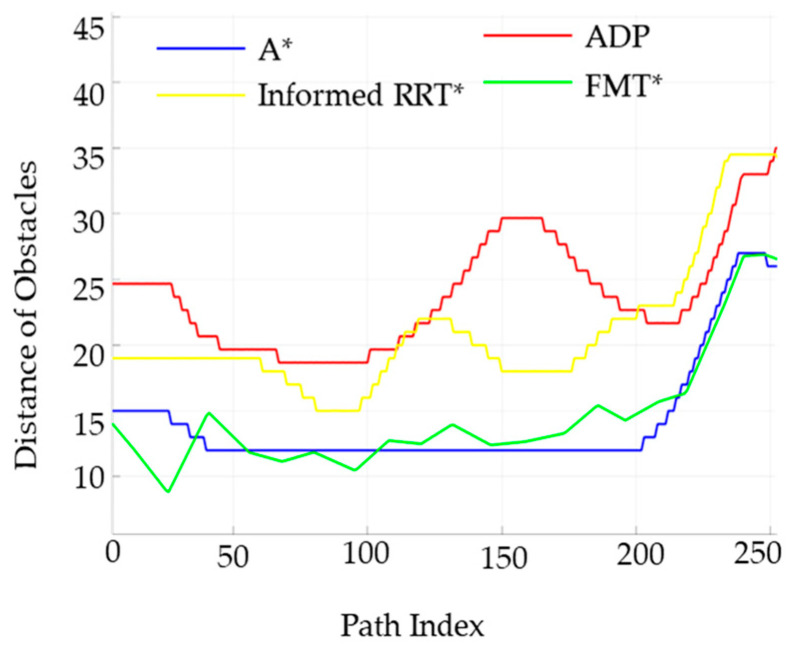
Comparison of obstacle distances between the four algorithms under low obstacle-density conditions.

**Figure 19:**
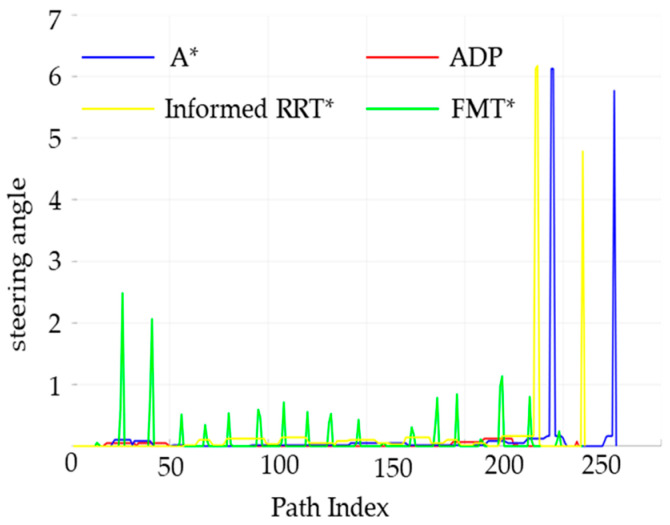
Comparison chart of steering angles for the four algorithms under low obstacle-density conditions.

The performance of the four algorithms in terms of key indicators such as obstacle distance values and angle-change values was analyzed, and the relevant results are shown in Figure 18 and Figure 19.

Figure 18 shows the distribution of obstacle distance values at different distances within the path range for the traditional A* algorithm, the FMT* algorithm, the Informed RRT* algorithm, and the ADP algorithm proposed in this paper. As can be seen from the figure, the algorithm proposed in this paper guarantees a larger obstacle distance value, indicating that it is safer without any restrictions.

The steering angle of the unmanned vehicle during the path-planning process, is shown in Figure 19. The steering-angle fluctuation of the ADP algorithm in this paper is significantly smaller than that of the other two algorithms, proving that the algorithm in this paper has better smoothness under low-density obstacles.

**Figure 20:**
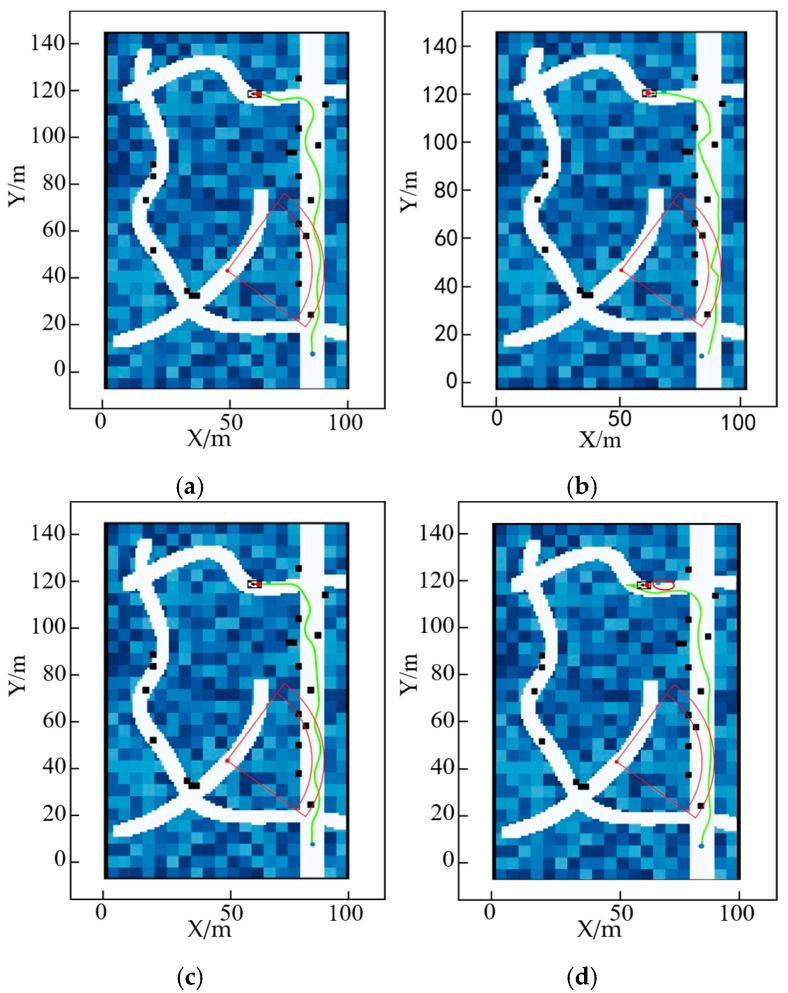
Comparison of planning results of different algorithms under high obstacle density: (**a**) A* algorithm; (**b**) FMT* algorithm; (**c**) Informed RRT* algorithm; (**d**) ADP algorithm.

To validate the effectiveness of the algorithm presented in this paper, we reduced the size of the obstacles while increasing their density and bringing the target point closer to the interference environment, as shown in Figure 20.

**Figure 21:**
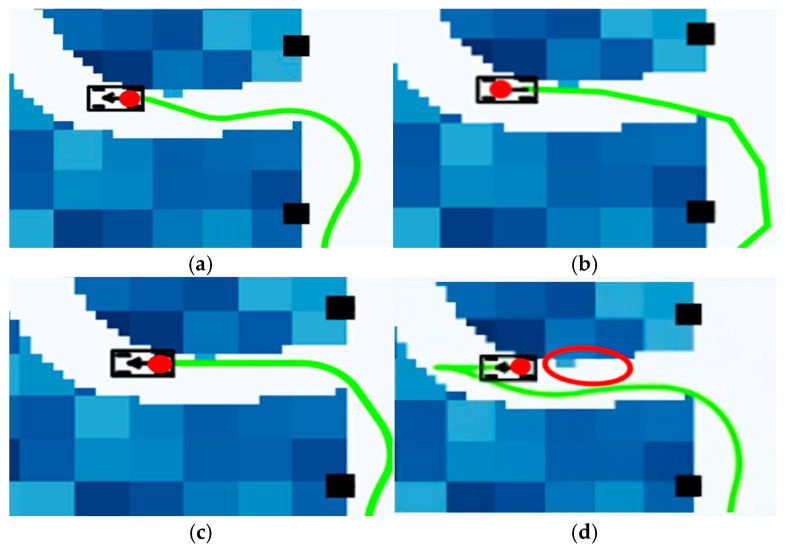
Local magnification of planning results for different algorithms under high obstacle density: (**a**) A* algorithm; (**b**) FMT* algorithm; (**c**) Informed RRT* algorithm; (**d**) ADP algorithm.

The results of the four different algorithms are shown in Figure 20 above. As can be seen from the figure, the traditional A* algorithm and the Informed RRT* algorithm have large steering angles, the FMT* algorithm has excessive steering, while the ADP algorithm proposed in this paper has a smoother path. The magnified view is shown in Figure 21. As can be seen from the figure, the algorithm in this paper effectively identifies the road surface that interferes with the chassis at the endpoint, as shown in the red circle. The experimental results of the four algorithms are compared in Table 3.

As can be seen from the table above, although the algorithm time is slightly higher than that for low obstacle density, the overall experimental results are similar to those for low obstacle density. In a high-density obstacle environment, the performance of the four algorithms was analyzed in terms of key indicators such as obstacle distance values and angle-change values.

**Figure 22:**
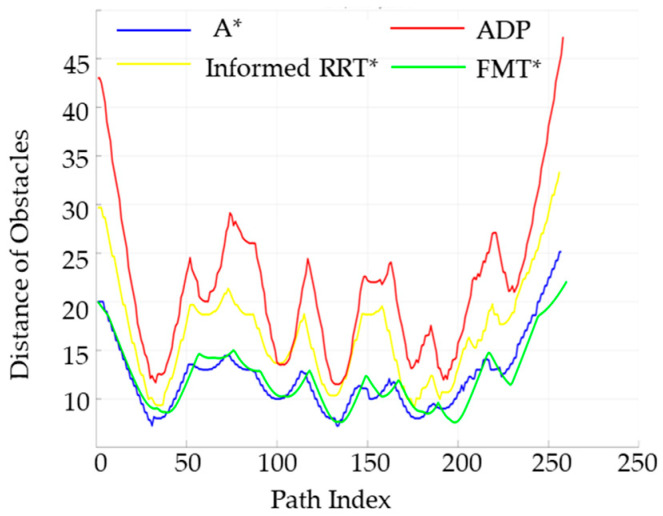
Comparison of obstacle distances between the four algorithms under high obstacle-density conditions.

**Figure 23:**
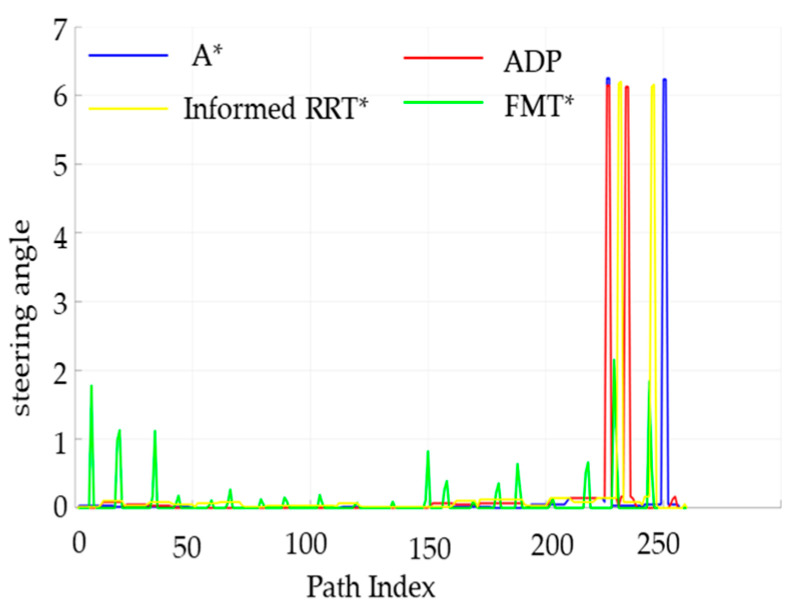
Comparison of four algorithms’ corner detection performance under high obstacle-density conditions.

As can be seen from Figure 22, in a dense obstacle environment, the algorithm presented in this paper still guarantees a large obstacle distance value, providing better safety. In a dense obstacle environment, the steering angle of the unmanned vehicle is shown in Figure 23.

As can be seen from Figure 23, the ADP algorithm proposed in this paper has the smallest fluctuation among the four algorithms, proving that the algorithm proposed in this paper has better smoothness under high-density obstacles.

**Figure 24:**
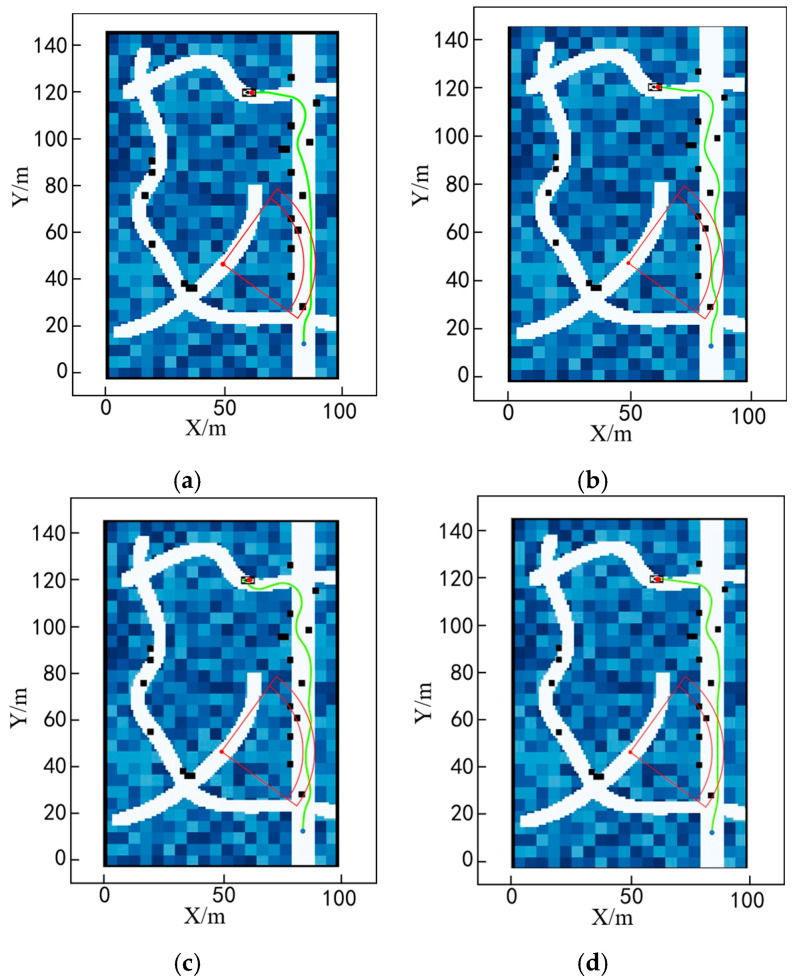
Path-planning results under different task modes: (**a**) Task Mode 1 path-planning results; (**b**) Task Mode 2 path-planning results. (**c**) Task Mode 3 path-planning results; (**d**) Task Mode 4 path-planning results.

To validate the effectiveness of the method used in this paper to dynamically output cost weights by fuzzy neural networks under different modes, and since the passage under dense obstacles may include cases under low-density obstacles, this paper only considers high-density obstacles and performs path planning for different task modes separately. The results are shown in Figure 24.

In Task Mode 1: Conveyance—its fuzzy characteristics are to stay away from danger points, follow a determined path, reduce jolts, and reduce frequent gear changes. Therefore, the generated path tends to be more linear, as shown in Figure 24a.

In Task Mode 2: Reconnaissance—its fuzzy characteristics are to approach dangerous points but not enter the danger zone, take low-risk routes, and not consider route length. Therefore, its path planning results are shown in Figure 24b.

Task Mode 3: Searching, with the following fuzzy characteristics—does not consider firing points, takes the path of least risk, does not consider path length, and does not consider direction changes. The planning results are shown in Figure 24c.

In Mission Mode 4: Drone takeoff and landing—its fuzzy characteristics are to travel in a straight line, control turbulence, and avoid obstacles. The planning results are shown in Figure 24d.

**Figure 25:**
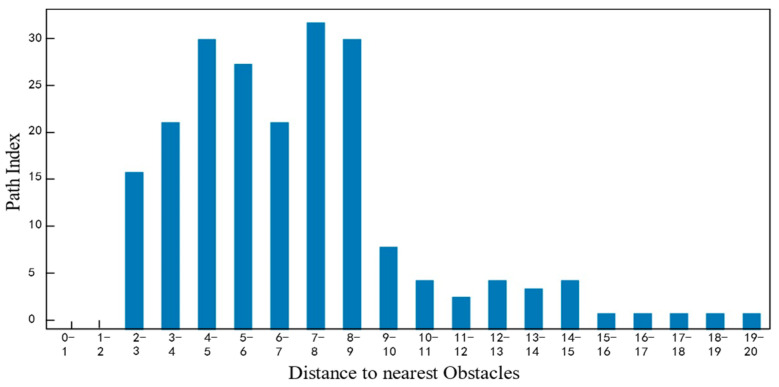
Task Mode 1: Obstacle distance statistics histogram.

**Figure 26:**
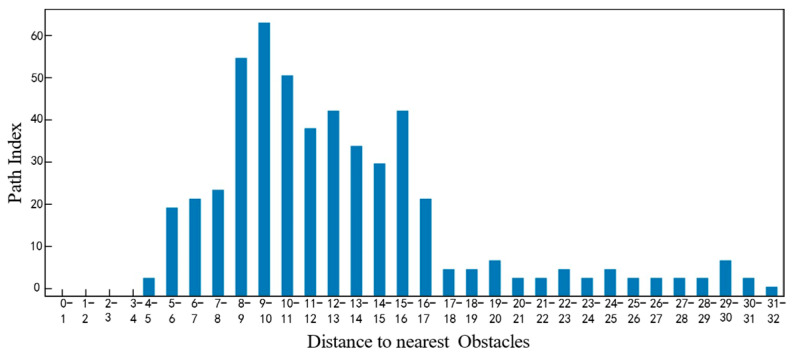
Task Mode 2: Obstacle distance statistics histogram.

**Figure 27:**
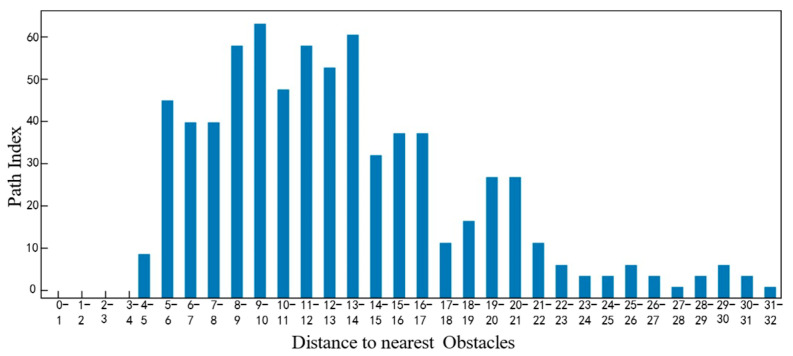
Task Mode 3: Obstacle distance statistics histogram.

**Figure 28:**
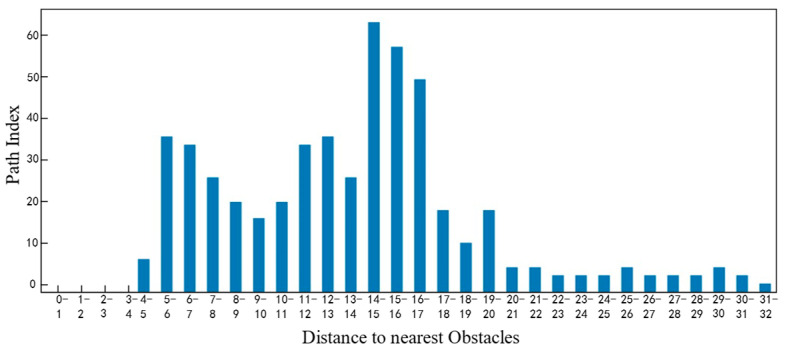
Task Mode 4: Obstacle distance statistics histogram.

As shown in Figure 24 above, by analyzing the current vehicle parameters and task characteristics, it is possible to generate a trajectory diagram that aligns with the task’s specific requirements. Figure 25, Figure 26, Figure 27 and Figure 28 show the statistical histograms of the distances between each node and the obstacles in the motion trajectory of the path-planning algorithm in this chapter. The horizontal axis represents the distance between each point on the path and the obstacle, while the vertical axis represents the number of points detected within that distance range. This further validates the effectiveness of the algorithm presented in this paper in the field of fuzzy neural network control.

## 5. Conclusions

This study proposes an adaptive path-planning method for UGVs in complex three-dimensional environments. This method constructs a three-dimensional map environment with terrain elevation information and an interference model for UGVs. It also proposes a mechanism that can dynamically adjust cost weights based on current environmental information during path planning, effectively improving path feasibility and environmental adaptability in unstructured terrain. On this basis, a multi-factor integrated cost function is constructed, and a task-driven dynamic programming strategy adjustment module is designed so that the generated path can respond in real time to path-planning requirements under different task conditions. Experiments were conducted to compare the path-planning results for UGVs in sparse and dense obstacle environments. The experimental results show that, compared with traditional A*, Informed RRT*, and FMT* path-planning algorithms, the method proposed in this paper has certain advantages in terms of obstacle-avoidance distance and trajectory smoothness. In addition, the paths planned by the algorithm in this paper were verified to be consistent with the characteristics of the corresponding tasks in four different task modes. In summary, the algorithm presented in this paper effectively improves the adaptability of UGV path planning in unstructured environments and its dynamic characteristics under different path-planning objectives.

## Figures and Tables

**Table 1:** Fuzzy control rules for path planning.

Fuzzy Rule Statement	Explanation of Meaning
High floor unevenness and low energy margins	Prioritize avoidance of bumps, not shortest paths
Low energy margin and low vehicle health	Poor health/power, need more secure path (not shorter but safer)
High vehicle health and low ground unevenness	Excellent condition, optimal path-seeking is the main focus
High density of obstacles and medium unevenness	Crowded and not very flat, with obstacle avoidance and stability prioritized
High energy margin in floor unevenness	Fair road conditions, enough energy, can accept appropriate detours and turns
High energy margin and low obstacle density	Reducing costs to reach goals as quickly as possible
High unevenness and high density of obstacles	Enhanced obstacle avoidance, stabilization, robustness requirements
Low ground unevenness and low vehicle health	Ground is good but the system is vulnerable to failure, choose the less bumpy paths
Low unevenness and high density of obstacles	Complex environment but good ground, allowing for flexibility
Low energy margin and high obstacle density	Common state, balanced planning

**Table 2:** Comparison of low-density environmental simulation test results.

Algorithm Category	Calculation Time (min)	Path Smoothness	Obstacle Distance (m)
A*	1.31 ± 0.08 (95%CI: ±0.04)	0.45 ± 0.05 (95%CI: ±0.02)	12.13 ± 0.05 (95%CI: ±0.26)
FMT*	0.56 ± 0.08 (95%CI: ±0.04)	1.48 ± 0.05 (95%CI: ±0.02)	8.75 ± 0.05 (95%CI: ±0.26)
Informed RRT*	2.05 ± 0.08 (95%CI: ±0.04)	0.33 ± 0.05 (95%CI: ±0.02)	15.14 ± 0.05 (95%CI: ±0.26)
ADP	1.45 ± 0.08 (95%CI: ±0.04)	0.21 ± 0.05 (95%CI: ±0.02)	18.25 ± 0.05 (95%CI: ±0.26)

**Table 3:** Comparison of high-density environmental simulation test results.

Algorithm Category	Calculation Time (min)	Path Smoothness	Obstacle Distance (m)
A* algorithm	1.47 ± 0.08 (95%CI: ±0.04)	0.59 ± 0.05 (95%CI: ±0.02)	5.14 ± 0.05 (95%CI: ±0.26)
FMT* algorithm	1.34 ± 0.08 (95%CI: ±0.04)	1.39 ± 0.05 (95%CI: ±0.02)	6.38 ± 0.05 (95%CI: ±0.26)
Informed RRT*	2.15 ± 0.08 (95%CI: ±0.04)	0.51 ± 0.05 (95%CI: ±0.02)	8.21 ± 0.05 (95%CI: ±0.26)
ADP algorithm	1.58 ± 0.08 (95%CI: ±0.04)	0.46 ± 0.05 (95%CI: ±0.02)	12.59 ± 0.05 (95%CI: ±0.26)

## Data Availability

Data will be made available upon request.
